# Rainbow Trout (*Oncorhynchus mykiss*) Pre-Smolts Treated with 11-Deoxycorticosterone Regulate Liver Carbohydrate Metabolism and Gill Osmoregulation

**DOI:** 10.3390/ijms26083725

**Published:** 2025-04-15

**Authors:** Rodrigo Zuloaga, Luciano Ahumada-Langer, Jorge Eduardo Aedo, Katalina Llanos-Azócar, Alfredo Molina, Juan Antonio Valdés

**Affiliations:** 1 Programa de Doctorado en Biotecnología, Facultad de Ciencias de la Vida, Universidad Andres Bello, Santiago 8370186, Chile; rodrigo.zuloaga.r@gmail.com (R.Z.); k.llanosazcar@uandresbello.edu (K.L.-A.); 2 Laboratorio de Biotecnología Molecular, Departamento de Ciencias Biológicas, Facultad de Ciencias de la Vida, Universidad Andres Bello, Santiago 8370146, Chile; lucianofranco.a@gmail.com (L.A.-L.); amolina@unab.cl (A.M.); 3 Interdisciplinary Center for Aquaculture Research (INCAR), Concepción 4030000, Chile; 4 Departamento de Biología y Química, Facultad de Ciencias Básicas, Universidad Católica del Maule, Talca 3466706, Chile; jaedo@ucm.cl

**Keywords:** DOC, mineralocorticoid receptor, glucocorticoid receptor, stress response, salmonid

## Abstract

Smoltification is stressful for salmonids, and cortisol is one of the central endocrine regulators for seawater adaptation. It has been established that cortisol plays both mineralocorticoid and glucocorticoid functions by MR and GR, respectively, since the aldosterone hormone is absent. Recently, investigations have proposed that the 11-deoxycorticosterone (DOC) mineralocorticoid precursor might support cortisol effects, but this mechanism remains unclear. Hence, we assessed the early effects of DOC on rainbow trout pre-smolts, the key smoltification stage, via metabolic and transcriptomic approaches. Thirty-six juveniles (~120 g) were treated for 3 h with DOC (1 mg/kg) and/or mineralocorticoid (eplerenone) or glucocorticoid (mifepristone) receptor antagonists (*n* = 6 for each group). DOC decreased plasma glucose and pyruvate and increased phosphate and liver glycogen. DOC also downregulated carbohydrate metabolism-related genes in the liver. Finally, gill RNA-seq analysis presented 1660 differentially expressed transcripts in DOC versus vehicle, 1022 for eplerenone + DOC versus DOC and 3324 for mifepristone + DOC versus DOC. The enrichment analysis mainly revealed the upregulation of ion transmembrane transport and carbohydrate metabolism and the downregulation of stress and innate immune responses. This suggests a significant role of DOC in liver carbohydrate metabolism and gill osmoregulation of pre-smolts through both receptors. Hence, this could contribute to improving animal welfare monitoring during smoltification by featuring novel and potential biomarkers.

## 1. Introduction

Intensive salmonid farming has become a significant industry worldwide [1,2]. Currently, fish production is carried out in two phases, which is in line with the natural life cycle of these fish [3]. During the first phase, juveniles of the parr development stage are cultured in freshwater or recirculating aquaculture systems (RASs) until they are ready to move to seawater cultures, later denominated the pre-smolt stage [4]. The process involving these changes is known as smoltification, which induces modifications in fish physiology and morphology to adapt from freshwater to seawater salinity [5]. Once the pre-smolt stage is confirmed (40 to 120 g), fish are transferred to marine cages to begin the fattening period until they are marketed [3,6]. 

However, intensive farming can negatively affect the growth and animal welfare of salmonids, mainly due to highly stressful conditions such as smoltification [7,8]. The stress response in fish starts with the activation of the hypothalamic–pituitary–interrenal (HPI) axis, followed by the elevated production of glucocorticoid hormones in the bloodstream, with cortisol being the main regulator [9]. In juvenile stages, cortisol has been described to participate in seawater adaptation [4,10] by regulating the hydromineral balance through changes in gill epithelial cell membrane permeability [11,12]. In addition, this hormone has been described to regulate carbohydrate metabolism, inducing glycogen degradation to generate glucose as an energy substrate and inhibiting its synthesis [13,14]. 

For several years, it has been theorized that cortisol performs both glucocorticoid and mineralocorticoid functions in fish as cortisol can bind to the glucocorticoid receptor (GR) and the mineralocorticoid receptor (MR) [15,16]. Interestingly, cortisol is the canonical ligand of the GR in mammals, and for the MR, it is aldosterone, a mineralocorticoid hormone that is not synthesized by fish [17]. The above suggests the existence of an agonist other than cortisol that could trigger different or complementary physiological functions. In this regard, recent data have evidenced that the aldosterone precursor hormone, 11-deoxycorticosterone (DOC), plays a physiological role as an MR ligand [17,18,19]. Like cortisol, DOC is synthesized from progesterone by the enzymatic activity of 21β-hydroxylase and is subsequently secreted by interrenal cells of the anterior kidney [20,21]. Previous studies suggest that DOC is involved in the early physiological responses of fish, affecting different tissues and biological processes [22,23,24,25,26,27]. In rainbow trout (*Oncorhynchus mykiss*), early-stage (parr) DOC induces a transcriptomic response for gill osmoregulation mainly through the MR [23], as well as increases the sodium–potassium pump (Na^+^ and K^+^ ATPase) α1-isoform expression in salmon (*Salmo salar*) gill explants [24]. Additionally, DOC increases glucose metabolism-related proteins in Eurasian perch (*Perca fluviatilis*) livers, as well as differentially regulates carbohydrate metabolism-related genes by the GR and MR in the liver of trout parr [25,26]. Finally, DOC has a significant effect on the transcriptomic response of trout parr skeletal muscle, which is differentially modulated by the GR and MR pathways and is complementary to cortisol [27]. 

Although the effects of DOC in the early salmonid stages have been previously studied [24,28,29], it is still unclear what role this hormone plays during the freshwater final phase, a critical step for proper smoltification. According to this, the questions about the distinct or complementary function of DOC regarding cortisol and whether DOC uses both the GR and MR pathways remain unanswered. We hypothesized that DOC could differentially modulate gill osmoregulation and liver metabolism in the pre-smolt stage using both corticosteroid receptor pathways. Therefore, we analyzed the early DOC-induced response in rainbow trout pre-smolt juveniles via metabolic, physiological, and transcriptomic approaches using both GR and MR pharmacological antagonists.

## 2. Results 

### 2.1. DOC and Cortisol in Plasma

**Figure 1:**
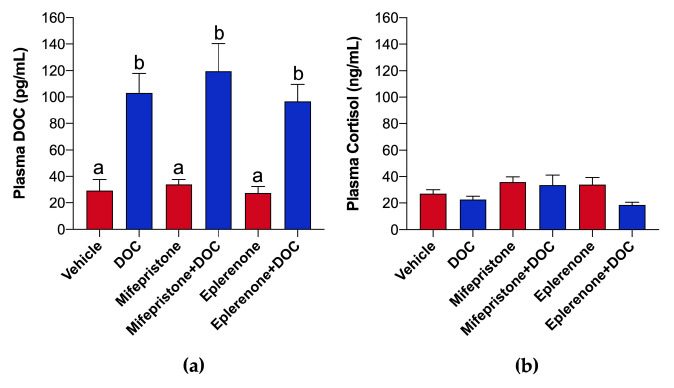
DOC, and cortisol quantification in plasma. DOC (**a**) and plasma cortisol levels (**b**) were quantified using MS and ELISA in pre-smolt juvenile fish treated with the vehicle, DOC, mifepristone, mifepristone + DOC, eplerenone, and eplerenone + DOC. The results are expressed as means ± SEM (*n* = 6 per group). Different letters represent significant differences at *p* < 0.01.

In plasma obtained from rainbow trout, significantly increased DOC levels were detected in the DOC-treated group (103 ± 14.95 pg/mL) vs. the vehicle group (29.34 ± 8. 34 pg/mL), mifepristone + DOC (119.5 ± 20.64 pg/mL) vs. mifepristone alone (34.04 ± 3.61 pg/mL), and eplerenone + DOC (96.73 ± 12.76 pg/mL) vs. eplerenone alone (27.59 ± 4.80 pg/mL) (Figure 1a). No significant changes in plasma cortisol concentrations were observed (Figure 1b).

### 2.2. Glucose, Lactzate, Pyruvate Plasma Levels, and Glycogen Content in Liver

**Figure 2:**
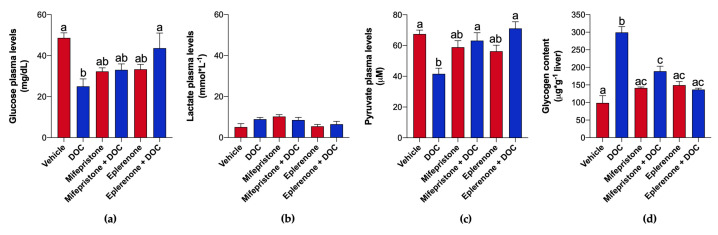
Detection of metabolites in plasma and liver glycogen. Plasma levels of glucose (**a**), lactate (**b**), and pyruvate (**c**); and liver glycogen content (**d**) was quantified using ELISA in the different groups of fish pre-treated with mifepristone or eplerenone incubated with the vehicle or DOC. The results are expressed as means ± SEM (*n* = 6 per group). Different letters represent significant differences at *p* < 0.01.

DOC-induced plasma glucose levels decreased significantly compared to the vehicle group (25.01 mg/dL ± 3.63; *p* = 0.0021; Figure 2a), which was reversed only when treated with eplerenone + DOC (43.66 mg/dL ± 7.351; *p* = 0.021), while no significant changes were observed when using the inhibitor mifepristone + DOC. Neither treatment resulted in significant changes in plasma lactate levels (Figure 2b). DOC compared to the vehicle group significantly decreased plasma pyruvate levels (41.59 ± 3.70 µM; *p* = 0.0012; Figure 2c), which was reversed when treated with mifepristone + DOC (63.17 ± 5.108 µM; *p* = 0.009) and eplerenone + DOC (71.11 ± 4.353 µM; *p* = 0.0002). As shown in Figure 2b, glycogen contents increased after DOC treatment vs. vehicle treatment (299.3 ± 16.43 μg·g^−1^; *p* < 0.0001), which were reversed upon treatment with both GR antagonist + DOC (188.9 ± 14.25 μg·g^−1^; *p* < 0.0001) and MR antagonist + DOC (137.0 ± 3.86 μg·g^−1^; *p* < 0.0001). 

### 2.3. Detection of Plasma Solutes and Muscle Water Content

**Figure 3:**
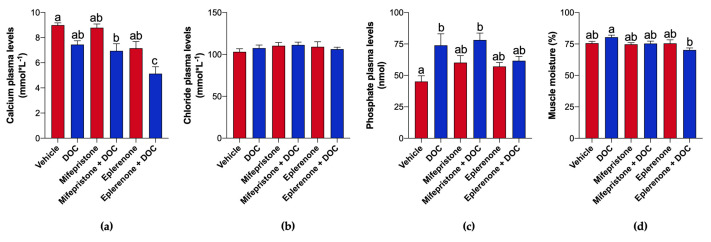
Quantification of solutes in plasma and the muscle water content. Plasma calcium (**a**), chloride (**b**), and phosphate (**c**) contents were measured using ELISA. Muscle water content (**d**) was quantified by measuring the difference between the wet weight versus dry weight of fish muscle. Results were obtained from rainbow trout treated with the vehicle, DOC, mifepristone, mifepristone + DOC, eplerenone, and eplerenone + DOC. Graphic are expressed as means ± SEM (*n* = 6 per group). Different letters represent significant differences at *p* < 0.01.

Plasma calcium levels decreased with DOC alone via MR (5.13 ± 0.55 mmol·L^−1^; *p* = 0.016, Figure 3a) compared to the DOC-only group; however, DOC treatment showed no significant change from the vehicle group. Plasma chloride levels did not show significant changes in all treatments (Figure 3b). While plasma phosphate levels increased significantly with DOC vs. the vehicle group (73.89 ± 9.20 nmol; *p* = 0.012), treatments with both receptor antagonists showed no changes (Figure 3c). The observed changes showed an increase in muscle moisture only between the eplerenone + DOC-treated group (70.19 ± 1.67%; *p* = 0.007) vs. DOC (Figure 3d). 

### 2.4. DOC-Induced Transcriptional Response Related to Carbohydrate Metabolism in the Liver

**Figure 4:**
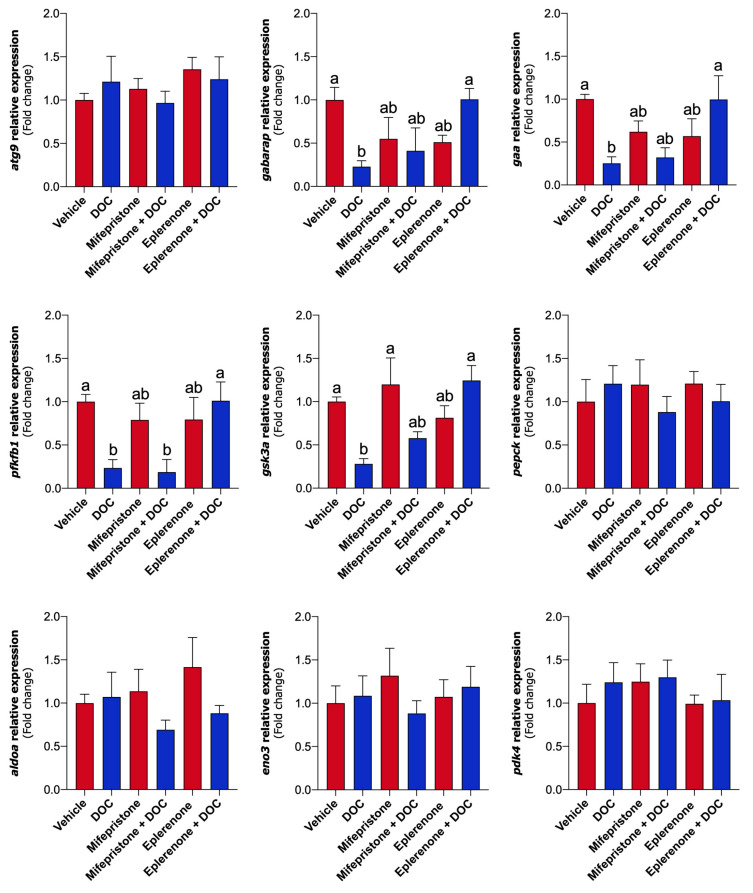
Transcriptional levels of genes regulated in pre-smolt livers by DOC via GR or MR. Key genes associated with glycofagia (*atg9*, *gabarap*, and *gaa*) and carbohydrate metabolism (*pfkfb1*, *gsk3a*, *pepck*, *aldoa*, *eno3*, *pdk4*) were evaluated via qPCR. Relative gene expression (fold change) was determined using the 2^−ΔΔCT^ method and normalized against *actβ* and *fau*. Data show the mean ± SEM of each group (*n* = 6). A one-way ANOVA statistical analysis was performed with Tukey’s post-test. Different letters indicate significant differences (*p* < 0.05). Gene abbreviations: autophagy-related protein 9 (*atg9*), GABA type A receptor-associated protein (*gabarap*), glucosidase alpha acid (*gaa*), 6-phosphofructo-2-kinase/fructose-2,6-biphosphatase 1 (*pfkfb1*), glycogen synthase kinase 3 alpha (*gsk3a*), phosphoenolpyruvate carboxykinase (*pepck*), aldolase, fructose-bisphosphate A (*aldoa*), enolase 3 (*eno3*), pyruvate dehydrogenase kinase 4 (*pdk4*), 40S ribosomal protein S30 (*fau*), and beta-actin (actβ).

The mRNA levels of the *gaa*, *gabarap*, *gsk3a*, and *pfkfb1* genes were significantly downregulated by DOC and upturned only via MR (Figure 4). No effects on *atg9*, *pepck*, *eno3*, *pdk4*, and *aldoa* were noticed in fish treated with DOC, mifepristone, or eplerenone plus DOC. Also, no transcriptional variations were detected by DOC through GR.

### 2.5. DOC Induces a Transcriptomic Response of the Rainbow Trout Gill Mediated by the Glucocorticoid Receptor (GR) and the Mineralocorticoid Receptor (MR) 

Results from the 18 cDNA libraries showed 1,571,455,430 total reads, of which 1,500,524,992 (94.49%) high-quality reads remained after the trimming step (Table S2). 

**Figure 5:**
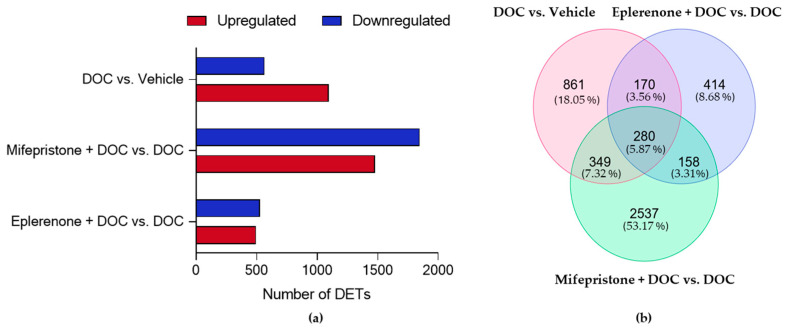
Differential expression analysis in pre-smolt gills. (**a**) Number of upregulated and downregulated expressed transcripts between the DOC vs. vehicle, mifepristone + DOC vs. DOC, and eplerenone + DOC vs. DOC groups and (**b**) Venn diagram indicating the numbers of common and unique differentially expressed transcripts in the three comparisons (padj < 0.05 and absolute Log2FC > 0).

The RNA-seq analysis of DOC vs. vehicle comparison showed 1660 DETs, with 1095 upregulated and 565 downregulated. In the mifepristone + DOC vs. DOC comparison, 3324 DETs were detected, with 1478 upregulated and 1846 downregulated (Figure 5a). Finally, in the eplerenone + DOC vs. DOC comparison, 1022 DETs were detected, with 494 upregulated and 528 downregulated. The complete list of DETs is in Table S3. From 4769 unique DETs in total, a Venn diagram analysis between the three comparisons detected 280 shared DETs, 861 unique for DOC vs. vehicle, 2537 for mifepristone + DOC vs. DOC, and 414 for eplerenone + DOC vs. DOC (Figure 5b).

**Figure 6:**
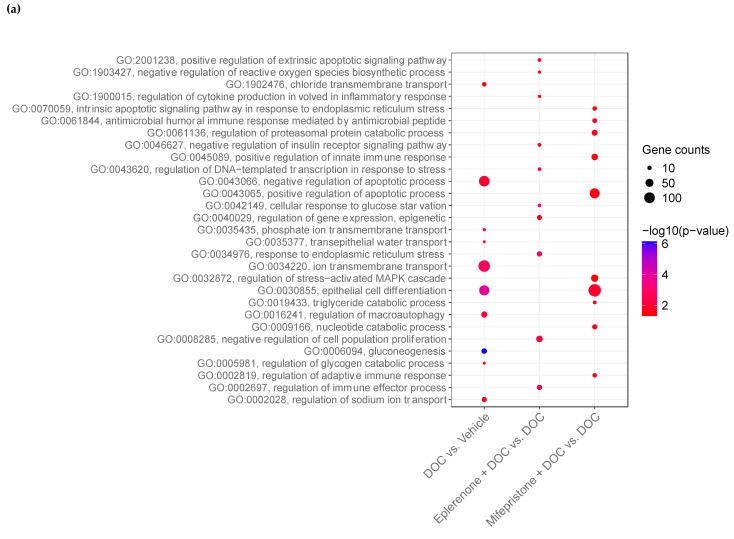

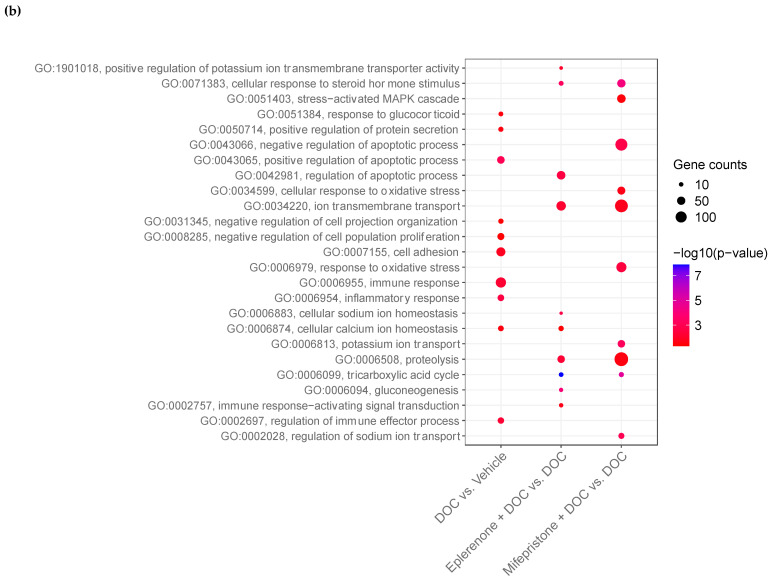
Gene enrichment analysis of biological processes in pre-smolt gills. The gene ontology biological process enrichment of the upregulated DETs (**a**) and downregulated DETs (**b**) in the DOC vs. vehicle, eplerenone + DOC vs. DOC, and mifepristone + DOC vs. DOC treatments. The results indicate the −log10(*p*-value) that was enriched in the differentially expressed transcripts with a padj of <0.05.

The data showed a significant difference in the biological processes associated with osmoregulatory processes, the stress response, carbohydrate metabolism, and processes associated with the innate immune response (Figure 6). Considering the upregulated DETs in the comparison of DOC vs. the vehicle, the biological processes related to osmoregulatory processes such as chloride transmembrane transport (GO:1902476), phosphate ion transmembrane transport (GO:0035435), transepithelial water transport (GO: 0035377), ion transmembrane transport (GO:0034220), and regulation of sodium ion transport (GO:0002028), as well as processes associated with carbohydrate metabolism such as gluconeogenesis (GO:0006094) and the regulation of the glycogen catabolic process (GO:0005981), were upregulated (Figure 6a). In the same comparison, downregulated DETs were identified as biological processes related to osmoregulatory processes such as cellular calcium ion homeostasis (GO:0006874), and the immune response (GO:0006955), the inflammatory response (GO:0006954), and the regulation of the immune effector process (GO:0002697) (Figure 6b). In the comparison between mifepristone + DOC vs. DOC, the biological processes associated with the antimicrobial humoral immune response mediated by antimicrobial peptides (GO:0061844), the regulation of the adaptative immune response (GO:0002819), the positive regulation of the apoptosis process (GO:0043065), and epithelial cell differentiation (GO:0030855) were upregulated (Figure 6a). Downregulated DETs included biological processes associated with osmoregulatory processes such as ion transmembrane transport (GO:0034220), potassium ion transport (GO:0006813), regulation of sodium ion transport (GO:0002028), proteolysis (GO:0006508), responses to oxidative stress (GO:0006979), and the cellular response to oxidative stress (GO:0034599) (Figure 6b). In the comparison between eplerenone plus DOC vs. DOC, the upregulated DETs were related to biological processes such as the regulation of cytokine production involved in the inflammatory response (GO:1900015), the negative regulation of the insulin receptor signaling pathway (GO:0046627), the cellular response to glucose starvation (GO:0042149), the regulation of the immune effector process (GO:0002697), and the negative regulation of cell population proliferation (GO:0008285) (Figure 6a). The downregulated DETs were related to osmoregulatory processes such as ion transmembrane transport (GO:0034220), cellular sodium ion homeostasis (GO:0006883), the positive regulation of potassium ion transmembrane transporter activity (GO:1901018), and cellular calcium ion homeostasis (GO:0006874); processes associated with carbohydrate metabolism such as gluconeogenesis (GO:0006094) and tricarboxylic acid cycle (GO:0006099); processes related to the cellular response to steroid hormone stimulus (GO:0071383); and immune response-activating signal transduction (GO:0002757) (Figure 6b). The total enriched biological processes, molecular functions, cellular components, and KEGG pathways between all comparisons are detailed in Table S4.

## 3. Discussion

This study presents new insights into the effects of DOC on rainbow trout in the final freshwater stage or pre-smolt, which is a critical stage for proper smoltification and determines that both the GR and MR pathways (using pharmacological antagonists) participate via transcriptomic and metabolic approaches. It first analyzed the DOC and cortisol plasma levels on pre-smolts to ensure that exogenous DOC induced all the effects. A significant rise in DOC plasma levels to 103 pg/mL was noticed in the DOC-treated fish compared to 29 pg/mL in the control. Using an equivalent strategy, a similar pattern was reported in the trout parr stage [27], which was comparable under physiological states. Based on this, DOC levels between 45 and 271 pg/egg were detected just after fertilization until the swim-up stage of rainbow trout [28]. In agreement with our findings, juvenile trout raised five-fold more DOC plasma levels (around 10 to 50 pg/mL) at 4 h of confinement stress [22]. Still, the DOC plasma levels can differ under stress conditions, development phases, and the species [29,30,31,32]. For instance, an increase in hepatic *gr* expression and a flow rate decrease were observed during a short DOC perfusion (10 nM) in North Pacific spiny dogfish (*Squalus suckleyi*) [19], and similar DOC concentrations were used in this study. By contrast, higher concentrations of DOC (4 mg/kg) were employed in Eurasian perch, which resulted in slightly increased plasma glucose levels compared to cortisol implants [25]. The differences could be related to the hormone dose used and the chronic and acute effects of DOC on the energetic physiology of lower vertebrates. However, the action of exogenous DOC could be affected by the elevated cortisol plasma levels. Previous studies indicated that cortisol presents 10- to 1000-fold more content in blood than DOC, but DOC can induce an MR transactivation 10 times higher than cortisol in vitro [18]. Therefore, a metyrapone pretreatment was included to reduce the activity of 11-hydroxylase which inhibits cortisol production [33]. Likewise reported in the parr stage [27], no significant changes or increases between all groups were found on plasma cortisol levels of trout pre-smolts. Yet, the low cortisol plasma levels detected (between 20 and 40 ng/mL) could be due to the handling during metyrapone administration and the sedation of fish. The evidence here suggests that DOC could have a role not only during early development stages but also during the final freshwater phase of juvenile salmonids.

The metabolic and transcriptional response was assessed next, evidencing new findings that DOC can also differentially regulate the liver carbohydrate metabolism of pre-smolts through both corticosteroid receptors. At the metabolic level, the glucose and the pyruvate in plasma and glycogen liver content were altered by DOC, mainly through MR, on rainbow trout pre-smolts. Although similar results were evidenced in the parr stage [26], only glucose plasma levels did not change [27]. When using the same dose in perch [25], treatment with another corticosteroid precursor (11-deoxycortisol) in sea lamprey (*Petromyzon marinus*) significantly raised the levels of glucose in plasma [31]. Since an equivalent strategy was used, these differences could be due to the development stage, but additional experiments are required. Nevertheless, it is known that cortisol induces glycogen breakdown to promote hyperglycemia after stressful conditions in the short term [34,35,36]. This complementary action could explain the DOC’s effects on carbohydrate metabolism, which decreases glucose and pyruvate plasma levels and increases glycogen stored in the liver. To support this, the expression of several genes related to carbohydrate metabolism in the liver was analyzed, resulting in a downregulated expression of *gaa*, *gabarap*, *gsk3a*, and *pfkfb1* mainly through MR. The *gabarap*, *atg9*, and *gaa* genes encode proteins of an alternative glycogenolysis process, called glycophagy [37,38]. Briefly, this mechanism involves the breakdown of glycogen stored inside lysosomes from the liver or muscle by the GAA enzyme. The process begins with starch-binding protein 1 (STBD1) activation, which recruits the synthesized glycogen to be stored inside autophagosomes, which will be recognized by the GABARAP receptor. The mature glycophagosome will fuse with GAA-containing lysosomes to degrade glycogen and release glucose into the bloodstream [39,40]. These results are in line with recent work in fish under the stress response [41,42], supporting the effects of DOC in specific pathways of carbohydrate metabolism. The regulation of this mechanism could also be due to the downregulated expression of *gsk3a*, a kinase that modulates several signaling pathways, but mainly the glycogen metabolism [43]. Still, glycogen synthesis or degradation is closely related to glucose metabolism [13,44], which DOC can also regulate. Although important genes related to glycolysis/gluconeogenesis were assessed (*aldoa*, *eno3*, *pepck*, and *pdk4*), DOC differentially regulated only the expression of *pfkfb1*. This gene encodes a bifunctional enzyme of glucose metabolism that catalyzes both fructose-2,6-bisphosphate degradation and synthesis [44]. The downregulation of *pfkfb1* by DOC though MR was previously detected in rainbow trout parr liver [26], suggesting similar effects on pre-smolts. This agrees with other reports in zebrafish (*Danio rerio*) that not only GR but also MR has a key function in carbohydrate metabolism [45]. Interestingly, none of the genes here were regulated by DOC through GR in pre-smolt liver. Although there is broad knowledge of cortisol/GR-induced catabolism [35,46], these data suggest that MR would have an anabolic role that could limit GR action [45]. This work presents new evidence of DOC’s actions on liver carbohydrate metabolism in the rainbow trout pre-smolt stage, primarily through MR that could complement cortisol effects.

Later, the physiological and gill RNA-seq results indicated new effects of DOC that differentially modulate gill osmoregulation through both the GR and MR pathways. In juvenile pre-smolts, the plasma levels of phosphate and calcium were affected by DOC. Compared to rainbow trout parr [26], the exogenous DOC injection increased the phosphate plasma levels that returned to basal concentrations using the MR antagonist, meanwhile calcium plasma levels were decreased in a synergic manner with both corticosteroid receptor inhibitors. To date, there is no research about the functions of these ions in fish plasma under the DOC effect. Hence, we speculate that DOC, such as cortisol, uses osmoregulatory mechanisms to control the ion flux. Previous studies reported that cortisol regulates the mineral metabolism of fish bone during stress via GR, affecting phosphorus/calcium homeostasis [47,48,49]. Moreover, cortisol can rapidly induce calcium release in various fish [50,51]. Based on this, the early activation of MR by aldosterone was related to quick alterations of ion transport in mammalian vascular tissue, including intracellular calcium concentrations [52]. On the other hand, chloride plasma levels remain unchanged, suggesting that DOC could play other effects compared to previously reported fish treated with cortisol [53,54]. Some chloride cotransporters were differentially expressed in pre-smolt gills (such as the cystic fibrosis transmembrane conductance regulator (cftr) or chloride channel protein 2 (clcn2)), like the parr stage [23]. In addition, we evidenced a significant decrease in water muscle content in eplerenone + DOC compared to the DOC group. This could be due to the transcriptional variations in the water regulator and transporter *aquaporin1* (*aqp1*), suggesting differential gill osmoregulation that depends on the freshwater stage of juvenile rainbow trout. To support this, a gill RNA-seq analysis was assessed, revealing several biological processes regulated by DOC via GR and MR, including the stress response, carbohydrate metabolism, innate immunity, and apoptosis, and highlighting osmoregulation. As expected, the stress response was differentially regulated by DOC, supporting its stress-related hormonal effects on pre-smolt gills as previously reported in parr [23]. Since DOC also regulates gill transcription related to apoptosis, innate immunity, and carbohydrate metabolism [23], this might denote more complex physiological responses and a tissue-specific effect during stress in the short term. However, further analyses are required to determine these effects on fish gills. The background has shown that DOC regulates the gene expression related to behavior, the immune response, reproduction, metabolism, and osmoregulation [21,25,30,55,56,57]. This modulation is mainly associated with the MR pathway since previous reports concluded that DOC cannot bind to GR1 or GR2 in fish [18,58]. This interaction is still in debate [15,59,60], but recent research indicated novel functions of DOC through both corticosteroid receptors [27]. Osmoregulation is an important process on gills that requires large amounts of energy during stress, where carbohydrate metabolism is relevant to supply these substrates [61,62]. In this sense, several investigations have demonstrated that cortisol plays a key role in fish’s salinity tolerance [63,64,65], but the function of DOC in these mechanisms is less comprehended. Like juvenile parr [23], the osmoregulation-related DETs discovered, which were induced by DOC in pre-smolt gills, were mediated through MR, as well as through GR. These are involved in paracellular ion transport (*claudin1* (*cldn1*), *claudin6* (*cldn6*), and *occludin* (*ocln*) of the tight junction proteins system), as well as the transcellular mechanism by ion transmembrane cotransporters (potassium two-pore domain channel subfamily K member 1 (kcnk1), solute carrier family 24 member 5 (slc24a5), and cftr) [11,66]. However, the following novel DETs appeared in pre-smolts, suggesting a differential effect of DOC in the later development phase in freshwater: *clcn2*, a chloride transmembrane cotransporter of epithelial cells [67,68]; *aqp1*, a transepithelial water transporter that regulates osmotic processes [69,70]; *sodium hydrogen exchanger 3b* (*nhe3b*), a sodium transmembrane cotransporter of epithelial cells [71,72]; *phospholemman* (*plm*), also known as *fxyd1*, an auxiliary and regulatory protein that modulates the Na^+^ and K^+^ ATPase activity [73,74]; and most interestingly the *sodium/potassium-transporting ATPase subunit alpha-1* (*nkaα1*) and *sodium/potassium-transporting ATPase subunit beta-233* (*nkaβ233*), subunit isoforms of the main gill osmoregulatory in fish and essential during smoltification [66,75]. Gill explants of salmon treated with DOC evidenced a significantly raised expression of α-1b and α-1a subunit isoforms of Na^+^ and K^+^ ATPase [24]. Also supporting these new results, DOC differentially regulated the expression of *nkaα1* and *Na^+^–K^+^–2Cl^−^ cotransporter 1* (*nkcc1*) in Mozambique tilapia (*Oreochromis mossambicus*) and striped bass (*Morone saxatilis*) [30]. Nevertheless, DOC does not regulate other well-known cotransporters of ions on gill rainbow trout explants, such as CFTR and NKCC [76]. Although previous reports of the mineralocorticoid functions of DOC in fish were discussed, which have a minor effect on osmoregulation [29,77], no data related to other transcellular cotransporters, additional tight junction proteins, or transepithelial water transporters were mentioned. Overall, this study presents the novel effects of DOC on gill osmoregulation in rainbow trout in the final freshwater or pre-smolt stage through both corticosteroid receptors that could complement cortisol effects.

This work has certain limitations, so the data should be analyzed with caution. For instance, the specificity and systemic effects of pharmacological antagonists for blocking MR or GR was not determined. Both eplerenone and mifepristone (RU486) have been formerly used to analyze these pathways in fish, including rainbow trout [29,34,45,78,79,80,81]. Nonetheless, we are aware that RU486 can also prevent the progesterone receptor from binding in teleost fish [82]. This effect is improbable because pre-smolts were sexually immature during trial (juvenile stage), which is also consistent with the data of juvenile *Neolamprologus pulcher* treated with cortisol [81]. Additionally, this experimental design clearly differs from ecologically or commercially relevant stress scenarios. While the aim here was to evaluate DOC’s action in the final freshwater stage and which GR and/or MR pathway triggered its early effects, this does not consider the role of cortisol and other stress regulators during natural or salmonid farming smoltification, such as temperature, photoperiod, and prolactin, among others [4,5,10]. Still, the employment of pharmacological experiments must be considered, and experimental setup improvements are necessary to guide future research directions.

## 4. Materials and Methods

### 4.1. Experimental Protocol

Thirty-six pre-smolt juvenile rainbow trout (mean weight 119.94 ± 5.34 g and length 20.29 ± 0.31 cm) were acclimatized and maintained at a temperature of 14 ± 1 °C under light conditions of a 12-h dark cycle and a 12-h light cycle for two weeks; they were fed daily with commercial pellets at 1.5% of their body weight (Skretting, Puerto Montt, LL, Chile); freshwater quality was monitored by pH (~7), total hardness (4–7 °dGH), carbonate hardness (3–6 °dKH), 0 mg/L of nitrite (NO_2_), nitrate (NO_3_), and chlorine (Cl_2_) (Aquavital Multitest, Aquarium Münster, Telgte, Germany). 

For the assay, fish were first separated into three groups of twelve fish per tank and then sedated with benzocaine (25 mg/L, #BZ^®^-20, Veterquimica, Santiago, RM, Chile) for 1 min. All groups were injected with an endogenous cortisol inhibitor, metyrapone (#M2696, Sigma-Aldrich, Burlington, MA, USA), at a dose of 1 mg per kilogram of fish into the abdominal cavity for 1 h to suppress cortisol effects. The second group were with the GR glucocorticoid receptor inhibitor, mifepristone (RU486, #M8046, Sigma-Aldrich), at a dose of 1 mg per kilogram for 1 h, and the third group was with eplerenone (#107724-20-9, Santa Cruz Biotech, Santa Cruz, CA, USA) at a dose of 1 mg per kilogram for 1 h. Then, the three groups were separated into two new tanks with six fish each and sedated again. Three tanks received 0.1% DMSO-1X PBS (vehicle as the control), and the others received 11-deoxycorticosterone acetate (DOC) (#56-47-3, USBiological, Salem, MA, USA) at a physiological concentration of 1 mg per kilogram of fish for 3 h. The dose and time utilized for all compounds were determined before adjusting to analyze early effects on rainbow trout juveniles [27,77,78,83].

### 4.2. Sampling Process

After all treatments (*n* = 6 each group), rainbow trout were euthanized using benzocaine at a concentration of 300 mg/L. The entire protocol was approved by the bioethics committee of University and Chilean (protocol code 012/2023), and we adhered to animal welfare procedures. Following euthanasia, blood samples were collected from the flow vessel using a 3 mL heparinized syringe (#9041-08-1, Santa Cruz Biotech, Santa Cruz, CA, USA, at 10 mg/mL). Plasma was obtained by centrifuging the blood for 10 min at 5000× *g*. Additionally, gills and liver were obtained from all fish and stored in RNA Save (#01-891-1, Biological Industries, Kibbutz Beit Haemek, Israel) for 24 h at 4 °C and then at −80 °C. Plasma and muscle tissues were flash-frozen with liquid nitrogen and stored at −80 °C.

### 4.3. DOC and Cortisol Detection in Plasma

The quantification of DOC in plasma was performed via mass spectrometry (MS) according to Milla et al., 2018 [25]. Concisely, 3 mL of cyclohexane/ethyl acetate, 500 µL of water, and 50 µL of plasma were mixed and centrifuged for 10 min at 3500× *g*, and the same procedure was performed on the solid phase. The combination of both fractions was then dried using the SpeedVac system, and 100 µL of methanol was added for resuspension. The LC/MS–MS analysis was performed using a Quattro Ultima Platinum triple quadrupole mass spectrometer with a Varian Polaris C18 column (Micromass, Manchester, UK). Methanol and acetic acid (0.1%) were employed for the mobile phases using standard parameters of MS. The quantification of cortisol in plasma was performed using a Cayman cortisol kit (Cayman Chemical, Ann Arbor, MI, USA) following the manufacturer’s instructions. The linear range of cortisol detection was between 6.6 and 4000 pg/mL. The kit was previously validated with rainbow trout [27,78,83].

### 4.4. Measurement of Metabolites in Plasma 

The quantification of glucose (#10009582; Cayman, MI, USA), lactate (#ab65331; Abcam, MA, USA), and pyruvate (#700470; Cayman, MI, USA) in plasma was realized following the manufacturer’s protocols. The linear range of glucose detection was 2.5–25 mg/dL. The linear range of lactate detection was 0.02–10 mM/well. The linear range of pyruvate detection was 0–150 µM/well. All metabolites were validated previously in several species of fish plasma, including rainbow trout [78,83,84].

### 4.5. Quantification of Glycogen in Liver Tissue

An enzyme assay kit for glycogen quantification (#700480, Cayman, MI, USA) in 100 mg of liver was used according to the manufacturer’s instructions. Detection was performed using a linear range of 0.031–2 μg/well. The kit was previously validated with different teleost tissues [85,86].

### 4.6. Quantification of Solutes in Plasma 

Plasma levels of calcium (Ca^+2^, #MAK022, Sigma-Aldrich), chloride (Cl^−^, #MAK023, Sigma-Aldrich), and phosphate (PO_4_^−^, #BML-AK111, BIOMOL^®^ Green, Enzo, NY, USA) were quantified using colorimetric assays according to the manufacturer’s instructions. The linear range of calcium detection was between 0.4 and 2 μg/well. The linear range of chloride detection was between 20 and 100 nmol/well. The linear range of phosphate detection was between 0.03 and 2 nmol/well. All solutes were previously evaluated and validated in plasma from other teleost fish [87,88,89].

### 4.7. Detection of the Muscle Water Content

From 100 mg of rainbow trout muscle, the tissue water content was measured following the protocol previously described by [23]. Briefly, the weighed tissue was incubated at 56 °C overnight and reweighed the next day. The difference between the wet and dry weights of the muscle tissue allowed the water content to be determined [90].

### 4.8. RNA Extraction

Total RNA from gill and liver was obtained from 100 mg of tissue using the EZNA^®^ Total RNA kit (#R6834-00S, OMEGA Bio-Tek, Norcross, GA, USA), following the manufacturer’s instructions. Subsequently, RNA was purified using the RNA Clean and Concentrator™-5 kit (with DNase I) (#R1013, Zymo Research, Orange, CA, USA), following the manufacturer’s instructions.

### 4.9. Real-Time PCR in Liver

The RNA obtained was quantified using a Nanodrop kit, and integrity was visualized via 1.2% agarose gel electrophoresis. Then, from 1 μg of RNA, reverse transcription was performed using the ImProm-II™ kit (#A3800, Promega, Madison, WI, USA). Real-time PCR (qPCR) reactions were performed using the AriaMx thermal cycler (Agilent, Santa Clara, CA, USA). qPCR was performed using a mixture of 7.5 μL of 2× Brilliant II SYBR^®^ master mix (#600828, Agilent), 0.75 μL of each primer (250 nM), and 6 μL of cDNA (diluted 20-fold) in a final volume of 15 μL. Reaction controls included (i) a non-reverse transcriptase control (noRT) and (ii) a negative control (NTC). All amplifications were performed under the following conditions: initial denaturation at 95 °C for 5 min, 40 cycles at 95 °C for 15 s denaturation, Tm for 15 s annealing, and 72 °C for 15 s elongation. Melting curve analysis was performed to determine a single PCR product. The relative gene expression was determined using the 2^−ΔΔCT^ method [91], and the data were expressed as times of change with respect to the control group (vehicle). The 40S ribosomal protein S30 (*fau*) and beta-actin (*actβ*) genes were used as normalizers. All primer information used is shown in Table S1.

### 4.10. Library Construction, RNA-Seq Analysis, and Functional Annotation Analysis in Gills

The quality of gill RNA was determined using an automated capillary electrophoresis Fragment Analyzer CE system (Advanced Analytical Technologies Inc., Ames, IA, USA). Total RNA quantity was determined using a fluorometer and a Qubit RNA BR assay kit (#Q10210, Invitrogen, Carlsbad, CA, USA). RNA samples with RQN values greater than or equal to 8 were selected for library construction. Eighteen libraries (*n* = 3 per group) of cDNA were generated with a TruSeq v2 RNA sample preparation kit (#RS-122-2001, Illumina, San Diego, CA, USA) from 800 ng of RNA. Libraries were then quantified with a Kapa library quantification kit (#kk4824, Roche, Basel, Switzerland) on an AriaMx real-time PCR (qPCR) thermal cycler (Agilent, Santa Clara, CA, USA), and their size was determined via capillary electrophoresis. The libraries generated were sequenced on the Novaseq 6000 platform (Illumina) at Macrogen (Seoul, Republic of Korea) using a paired-end strategy (2 × 150 bp).

The quality of the raw reads was determined with MultiQC v1.26 [92]. Subsequently, the Fastp v.0.24 software [93] was used to discard adapters, low quality reads (Q < 30), low complexity filters (y), and poly X trimming in the 3′ ends. Then, using Kallisto v.0.51.1 [94], the high-quality reads were mapped and counted (default parameters) against the rainbow trout reference genome USDA_OmykA_1.1 (RefSeq GCF_013265735.2). The identification of differentially expressed transcripts (DETs) was performed using the R package DESeq2 v1.46 [95], with padj < 0.05 and absolute Log2FC > 0. Potential DOC-regulated DETs were detected in comparison with the DOC vs. vehicle groups. To determine potential DOC-regulated DETs via GR or MR, comparisons were made between mifepristone + DOC vs. DOC and eplerenone + DOC vs. DOC. Given that the reference transcriptome of rainbow trout is poorly and incompletely annotated, a custom annotation with the eggNOG 5 database was generated using eggNOG-mapper v2.1.12 [96]. The DETs’ IDs were used to perform ontological enrichment analysis using topGO v2.58 [97]. Subsequently, a topGO object was generated in R software v.4.4.2, and enriched biological processes, cellular components, and molecular functions of each comparison were detected using Fisher’s test. Data representation was performed using the R package ggplot2 v3.5.1 [98]. Lastly, the enriched KEGG pathways were analyzed via DAVID v2024q4 using standard parameters [99].

### 4.11. Statistical Analysis

A normal (Gaussian) distribution test and a Kolmogorov–Smirnov normality test were used to analyze the data. Subsequently, one-way analysis of variance (ANOVA) followed by Tukey’s honest significant difference test was performed. Data in graphs were presented as the mean ± standard error of the mean (SEM) using GraphPad Prism v8 software (CA, USA). The minimum statistical significance was *p* < 0.05.

## 5. Conclusions

Aquaculture can be a crucial nutrition source for worldwide consumption in the future. Nevertheless, intensive culture conditions (smoltification and seawater transfer) induce stress in fish, altering their physiology and metabolism and hence affecting their growth and flesh quality. Since cortisol is considered the main neuroendocrine regulator of stress, few studies have evaluated the function of other hormones that could complement cortisol effects, such as DOC. In conclusion, this work demonstrates a novel hormone related to the stress response in rainbow trout pre-smolts. DOC differentially regulates the early metabolic, physiological, and transcriptional responses related to liver carbohydrate metabolism and gill osmoregulation through GR or MR. This information will contribute to comprehending other biological actions of DOC in fish, complementing cortisol. Also, it could be useful to improve animal welfare monitoring by incorporating novel and potential stress molecular biomarkers for salmonid farming, especially during the fish transfer from freshwater to seawater.

## Data Availability

The raw read sequences acquired from sequencing were deposited in the Sequence Read Archive (SRA) under the following accession numbers: BioProject PRJNA1222082; BioSamples SAMN46760837, SAMN46760838, SAMN46760839, SAMN46760840, SAMN46760841, SAMN46760842; and SRA SRR32304861, SRR32304862, SRR32304863, SRR32304864, SRR32304865, and SRR32304866. Data obtained and analyzed during this study will not be publicly available due to ethical or privacy restrictions but can be requested from the corresponding author.

## Supplementary Materials

The following supporting information can be downloaded at: https://www.mdpi.com/article/10.3390/ijms26083725/s1.

## Abbreviations

The following abbreviations were used in this manuscript:

DOC	11-deoxycorticosterone
MS	Mass spectrometry
ELISA	Enzyme-linked immunoadsorption assay
GR	Glucocorticoid receptor
MR	Mineralocorticoid receptor
DETs	Differentially expressed transcripts
SEM	Standard error of the mean
ANOVA	One-way analysis of variance
qPCR	Real-time PCR
GO	Gene ontology
RQN	RNA quality number
padj	*p* value adjust
